# Young adults’ access to mental health care: integrating the Primary Care Behavioral Health model into routine care in Sweden

**DOI:** 10.1186/s12913-025-13903-2

**Published:** 2026-01-16

**Authors:** Per Nilsen, Lise Bergman Nordgren, Kristin Thomas, Erica Skagius Ruiz, Hanna Israelsson Larsen

**Affiliations:** 1 Department of Health, Medicine and Caring Sciences, Division of Society and Health, Linköping University, Linköping, Sweden; 2 Department of Medicine, Faculty of Medicine and Health, Örebro University, Örebro, Sweden; 3 Division of Psychiatry, Region Örebro, Örebro, Sweden; 4 Division of Prevention, Department of Health, Medicine and Caring Sciences, Rehabilitation and Community Medicine, Linköping University, Linköping, Sweden; 5 Primary Health Care Centre, Cityhälsan Centrum, Linköping, Sweden

**Keywords:** Mental health, Primary Care Behavioral Health (PCBH), Young adults, Access, Interrupted time series

## Abstract

**Background:**

Mental health issues among young adults are increasing. Swedish data indicate a three- to fourfold increase in symptoms of anxiety and distress over the past two decades. Access to mental health services in primary care is often hindered by long wait times and resource limitations. The Primary Care Behavioral Health (PCBH) model, characterized by brief, team-based interventions delivered by behavioural health consultants (BHCs), offers a promising approach to improve access. However, limited evidence exists on its impact on young adult populations.

**Methods:**

A pragmatic stepped-wedge cluster trial was used to evaluate the impact of facilitating PCBH integration on young adults aged 18–24 years in nine primary care centres in Region Östergötland, Sweden. Data were collected from medical records spanning three phases: pre-facilitation (year 1), facilitation (year 3) and post-facilitation (year 4). Year 2 measurements were excluded to avoid contamination because primary care centres were receiving information about the PCBH model during that period. This ensured that pre-facilitation data reflected conventional primary care conditions. Four outcomes were investigated: (1) number of unique patients, (2) number of BHC appointments by all patients, (3) average waiting times for all appointments, and (4) average waiting times for first appointments. The study was partially carried out during the COVID-19 pandemic. While the pre-facilitation phase occurred before the pandemic, the facilitation phase partially overlapped with it and the post-facilitation phase took place entirely during the pandemic.

**Results:**

The number of unique young adult patients accessing BHC services increased by 84% during facilitation (*p* < 0.001) and remained 25% above baseline post-facilitation. Total BHC appointments increased by 16% during facilitation (*p* < 0.001) and 13% post-facilitation (*p* = 0.006). The steepest increase was in remote consultations. Average waiting times for all appointments decreased from 14.1 to 11.8 days during facilitation (*p* < 0.001), and reductions were sustained post-facilitation (13.4 days, *p* = 0.007). In-person first appointment waiting times also decreased significantly during and after facilitation (both 7.1 days versus 8.8 days at baseline; *p* < 0.001).

**Conclusions:**

Integrating the PCBH model into routine primary care improved access to mental health services for young adults, as demonstrated by increased utilization and reduced waiting times. The findings highlight the model’s potential for scalable, team-based mental health delivery in primary care. However, decreases post-facilitation suggest that sustained implementation support may be important for long-term impact. It is important to note that the lack of a comparison group precludes definitive conclusions regarding the effects of the model, as alternative explanations for the observed changes cannot be ruled out.

## Introduction

Mental health concerns are increasingly prevalent in contemporary society, posing a significant and escalating public health challenge [1–3]. Among these concerns, the increase in mental health issues among young adults is particularly striking [4]. In Sweden, the incidence of anxiety and distress among young adults has surged three- to fourfold over the past two decades [5]. Recent reports indicate that the COVID-19 pandemic has further exacerbated this negative trajectory [6, 7], especially affecting young women; almost a third are experiencing anxiety [6]. These mental health struggles can significantly impede individuals’ functioning, hinder their daily activities, strain social relationships and cause considerable distress to both young individuals and their families [2, 4, 8].

Primary care traditionally caters to young adults with mental health issues. However, several barriers hinder access to mental health care within conventional primary care settings. A high influx of patients seeking care and shortages of mental health professionals have resulted in restricted care opportunities, prolonged waiting times, delays and the need for multiple contacts before obtaining appropriate care [9–12]. These issues can have a significant impact on young individuals in both the short and long term; extended waiting times might decrease young adults’ engagement when they finally receive care, reducing the likelihood that treatment will be completed as intended [3, 4, 13]. Hence, there is an increasing demand for improved service delivery models dedicated to managing mental health issues among young adults.

Most of the mental health issues addressed in primary care settings can be managed effectively through psychological interventions or even self-help approaches [3, 14]. The Primary Care Behavioral Health (PCBH) service delivery model has shown promise to support mental health in primary care [15, 16]. PCBH has been described as a patient-centred service delivery model using a multi-professional team-based approach. The PCBH model constitutes a systematic reorganization of primary care services to more effectively integrate behavioral health within routine clinical practice. By embedding Behavioral Health Consultants (BHCs) into primary care teams and strategically utilizing existing human resources, the model enhances both accessibility and operational efficiency. Furthermore, it strengthens the capacity of primary care providers to manage behavior-related health concerns through structured interdisciplinary collaboration and the dissemination of shared clinical expertise.

The model emphasizes the importance of addressing mental health concerns at an early stage [17–19]. A fundamental principle of the PCBH model is to provide easy access to an on-site BHC. The BHC acts as a resource for the primary care staff and quickly arranges appointments for patients who express mental health concerns [20, 21]. A BHC in Sweden is typically a psychologist, psychotherapist or health care counsellor, who is usually connected to a primary care centre (PCC). The aim of the PCBH model is to accommodate appointments directly or within a few days after contacting primary care. The overarching goal is to improve mental health in primary care [17]. This objective is closely aligned with the overarching primary care goals; therefore, the model is both clinically and conceptually appealing.

Although the PCBH model has received increasing recognition, a critical gap remains in research evaluating its integration into routine primary care, particularly regarding its impact on young adult populations. Existing studies have focused on specific health conditions or narrowly defined groups, limiting generalizability. There is a need for comprehensive research that examines how the PCBH model influences access to mental health services for the broader population of young individuals within primary care settings. In response to this need, this study aims to evaluate the impact of the PCBH model in Sweden on the utilization of mental health services provided by BHCs to young adults aged 18–24 years and on waiting times for BHC appointments.

These findings are critical from both clinical and scientific perspectives because they respond directly to the increasing demand for timely and effective mental health care in primary care settings. Furthermore, the study offers valuable insights into the adaptability of the PCBH model across diverse health care contexts, with particular relevance for improving young adult mental health service delivery.

## Methods

### Study design

A pragmatic stepped-wedge cluster trial was used to evaluate the impact of the PCBH model on young adults’ access to mental health services. Access to the nine participating primary care centres working according to the PCBH model was assessed at three different time points.

### Research setting

**Fig. 1:**
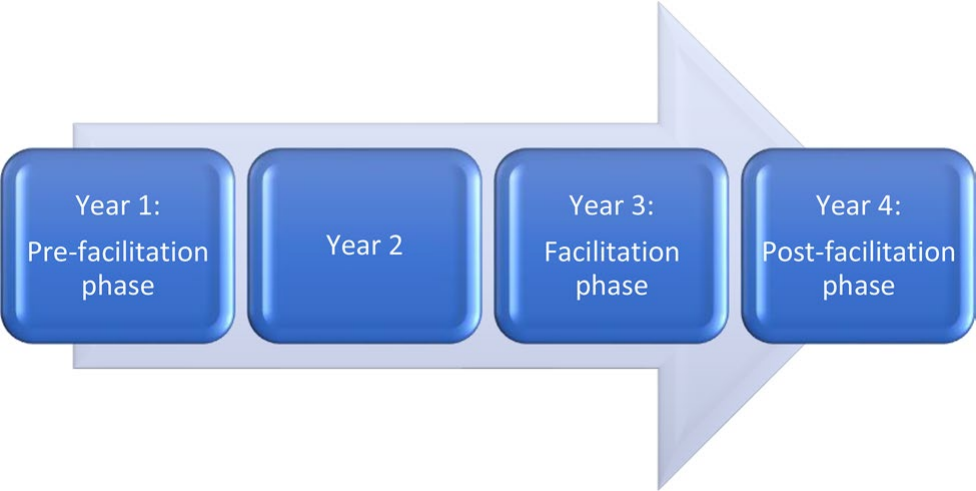
Three measurement phases over 4 years

The nine participating primary care centres were located in both urban and rural areas within Region Östergötland in southeastern Sweden. Region Östergötland, with a population of 471,912 in 2022, is the fourth most populous of Sweden’s 21 regions. The PCBH model was introduced at these centres according to a phased timetable. Accessibility was evaluated using a before-and-after design over three measurement periods (Fig. 1):

- Pre-facilitation phase (year 1): the year before the introduction of the PCBH model at each primary care centre;
- Facilitation phase (year 3): the year during which the model was introduced and facilitated by an implementation team; and.
- Post-facilitation phase (year 4): the year after the introduction of the model.

The decision to exclude measurements during year 2 was made to avoid potential contamination because the primary care centres were receiving information about the PCBH model during that time. By omitting measurements during the second year, we ensured that the pre-facilitation phase measurements represented conventional primary care when the PCBH model was introduced.

Swedish health care is predominantly government-funded; it is financed primarily through taxation and provides universal coverage for all citizens. Private health care is also available and, when publicly contracted, can be reimbursed through the national health system. The Ministry of Health and Social Affairs, supported by national government agencies, oversees health care policy and provides high-level guidance. However, the responsibility for funding, organizing and delivering health care is delegated to Sweden’s 21 regions. As such, most primary care centres and nearly all hospitals are regionally owned and operated. All residents are recommended to register with a specific primary care centre, in the region where they are listed as patients. This system promotes continuity of care and activates the statutory national guaranteed access to health care (i.e. that a citizen has the right to receive care within a certain period of time).

Information on the participating primary care centres is presented in Table 1: time periods corresponding to the three phases, the total number of listed patients, the total number of employees, BHCs and physicians.

**Table 1:** Characteristics of the nine participating primary care centres

	Pre-facilitation phase (year 1)	Facilitation phase (year 3)	Post-facilitation phase (year 4)	Total number of listed patients	Total number of employees
1	1 May 2017 to 30 Apr 2018	1 May 2019 to 30 Apr 2020	1 May 2020 to 30 Apr 2021	Year 1: 14,676	Year 1: 55
				Year 3: 14,768	Year 3: 58
				Year 4: 15,033	Year 4: 60
2	1 May 2017 to 30 Apr 2018	1 May 2019 to 30 Apr 2020	1 May 2020 to 30 Apr 2021	Year 1: 6733	Year 1: 19
				Year 3: 6880	Year 3: 20
				Year 4: 6993	Year 4: 20
3	1 May 2017 to 30 Apr 2018	1 May 2019 to 30 Apr 2020	1 May 2020 to 30 Apr 2021	Year 1: 8886	Private; information not available
				Year 3: 9014	
				Year 4: 9129	
4	1 May 2017 to 30 Apr 2018	1 May 2019 to 30 Apr 2020	1 May 2020 to 30 Apr 2021	Year 1: 6189	Year 1: 27
				Year 3: 6440	Year 3: 27
				Year 4: 6477	Year 4: 28
5	1 Dec 2017 to 30 Nov 2018	1 Dec 2019 to 30 Nov 2020	1 Dec 2020 to 30 Nov 2021	Year 1: 11,261	Year 1: 45
				Year 3: 11,776	Year 3: 43
				Year 4: 11,961	Year 4: 44
6	1 Dec 2017 to 30 Nov 2018	1 Dec 2019 to 30 Nov 2020	1 Dec 2020 to 30 Nov 2021	Year 1: 13,884	Year 1: 39
				Year 3: 14,326	Year 3: 46
				Year 4: 13,268	Year 4: 46
7	1 Dec 2017 to 30 Nov 2018	1 Dec 2019 to 30 Nov 2020	1 Dec 2020 to 30 Nov 2021	Year 1: 11,574	Year 1: 36
				Year 3: 12,343	Year 3: 38
				Year 4: 12,429	Year 4: 42
8	1 Dec 2017 to 30 Nov 2018	1 Dec 2019 to 30 Nov 2020	1 Dec 2020 to 30 Nov 2021	Year 1: 15,793	Year 1: 55
				Year 3: 15,683	Year 3: 56
				Year 4: 15,599	Year 4: 60
9	1 Dec 2017 to 30 Nov 2018	1 Dec 2019 to 30 Nov 2020	1 Dec 2020 to 30 Nov 2021	Year 1: 14,080	Year 1: 46
				Year 3: 14,446	Year 3: 49
				Year 4: 14,552	Year 4: 49

Data concerning listed patients and staffing were retrieved from local health care registries at the end of December for each phase.

### Facilitating the PCBH model

The PCBH model is a systematic reorganization of primary care delivery that enhances the integration of behavioral health into routine practice by embedding behavioral health consultants within primary care teams, thereby optimizing existing human resources and improving accessibility and efficiency. Integration of the PCBH model into routine operations at the nine primary care centres was facilitated by a regional implementation team of psychologists who had received specialized training in the PCBH model. The process of integrating the model adhered to the Swedish Public Health Agency’s “Checklist for Implementation with Quality” [22], an implementation framework based on the widely recognized Quality Implementation Framework (QIF). The QIF, as outlined by Nilsen [23], provides a structured approach to effective implementation, identifying 14 key activities across four critical stages: (1) initial assessment of needs and resources; (2) establishment of structures for implementation; (3) actual implementation; and (4) continuous learning and improvement [22, 24]. This systematic approach ensured a comprehensive and quality-focused integration of the PCBH model into the routine operations at the primary care centres.

At the start of the facilitation, each participating primary care centre formed a local implementation team comprising representatives from all key occupational categories, including at least one physician, nurse, manager and administrator as well as all BHCs. This local team acted as the liaison and discussion partner with the regional implementation team, handling communication and coordination. However, the local teams were not directly responsible for executing the facilitating activities; their role was primarily of an advisory and supportive nature, ensuring that the model remained aligned with the needs and resources of their specific centre.

After conducting a thorough needs and resource assessment at each participating primary care centre, the staff were provided with customized PCBH training, varying from 1 to 4 days depending on their professional role (Table 2). This targeted education was designed to equip each staff member with the necessary skills based on their specific responsibilities within the PCBH model. After the training, the regional implementation team crafted an action plan tailored to each centre’s organizational structure and requirements. The plan’s activities were then refined in collaboration with the local implementation teams to ensure they were adapted to the unique context of each primary care centre.

**Table 2:** Profession-specific education and supervision

	Nurses	Physicians	Behavioral Health Consultants
Education			
Time	2 days	1 day	4 days
Content	The PCBH model; the nurse’s professional role within the PCBH model; assessment and treatment of patients with behavioural and mental health problems; self-care advice for behavioural and mental health problems	The PCBH model; the physician’s professional role within the PCBH model; interview methodology for focused assessments of behavioural and mental health problems; behavioural perspective on mental illness; how to motivate patients to behavioural changes	The PCBH model; the behavioural health consultant’s professional role within the PCBH model according to the GATHER acronym (Generalist, Accessible, Team-based, High productivity, Education, Routine ) [17]; focused appointments; brief interventions
Supervised workshops and tutorials			
Time	1 h on three occasions (e.g. 1 h every 3 months)	1 h on three occasions (e.g. 1 h every 3 months)	1–2 h every month
Content	Discussions regarding clinical cases, adherence to core competences of the PCBH model and general questions/opinions related to the work with the PCBH model		

The minimum activities to facilitate the integration of the Primary Care Behavioral Health (PCBH) model into routine operations are listed. Additional support was provided according to the needs of each centre.

In the next step, concrete changes were carried out, including setting up BHC schedules and patient appointments. Working materials were distributed to the centre, e.g. checklists, new appointment templates, self-help material for patients and templates for internal procedures. While the implementation was ongoing, nurses, physicians and BHCs continuously supervised workshops and tutorials for each professional category at their respective centre (Table 2).

The regional implementation team had full responsibility for leading all activities to facilitate integration of the PCBH model into routine operations across the participating primary care centres. The facilitation phase at each centre lasted approximately 12 months, during which the regional implementation team closely guided the process. After this phase, additional support remained available upon request in the post-facilitation phase. The regional team offered ongoing, tailored support to each centre for at least 24 months, ensuring all centres received continuous guidance and necessary adjustments to facilitate the successful integration of the PCBH model into routine operations.

### Contextual adaptions of PCBH to a Swedish context

In standard primary care in Sweden, mental health therapists are already integrated within, or closely connected to, all PCCs. However, unlike BHCs, they do not typically serve in a consultative role. The first point of contact with a PCC is usually through a phone call or a secure text message. These are received by a nurse, who then triages the case and directs the individual either to an appointment with a nurse or with a physician. When a patient presents with mental health concerns, a written referral is typically sent to the mental health therapists at the centre. These therapists hold weekly referral meetings to determine whether the patient should be offered an appointment. If care is deemed necessary based on the written information, the patient is usually placed on a waiting list. Physicians can also refer patients directly by submitting a written request to the therapists.

The PCBH model was adapted to fit the Swedish healthcare context. Given the central role of nurses in initial patient assessment and triage, a key modification involved the development profession-specific educational materials and training programs, with a primary focus on nursing staff. This initiative enabled nurses to identify patients presenting with mental health concerns and to refer them for a BHC appointment at the initial point of contact.

Since long waiting lists for mental health services were identified as a major concern, stakeholders decided that the PCBH approach in Sweden would be applied exclusively to individuals presenting with behavioural or mental health problems, thereby excluding interventions primarily targeting somatic conditions.

Within this framework, the clinical workflow and scheduling of BHCs were reorganized. Both triage nurses and physicians were granted the ability to book patient appointments directly with BHCs, replacing the previous system of internal written referrals. In addition, BHCs were allocated dedicated time for direct consultation with both nurses and physicians.

In addition, broad adjustments were made regarding the use of focused BHC sessions. Because Swedish primary care is legally required to provide evidence-based treatment for mild to moderate psychiatric disorders, BHCs were expected to offer extended sessions when clinically indicated, in addition to the brief 15–30 min visits emphasized in the original model for accessibility and efficiency. As a result, most participating centres replaced the uniform 45–60 min standard visit with a more flexible appointment structure that allowed for both short and extended consultations.

### Participants

The inclusion criteria were patients aged 18–24 years who, at an appointment with a BHC or physician, had received any of the following ICD (International Statistical Classification of Diseases and Related Health Problems) diagnoses, regardless of the original reason for seeking care: F00–F99 (mental and behavioural disorders); Z56 (problems related to employment); Z73 (problems related to life-management difficulties). The study also included all patients aged 18–24 years who had been prescribed any psychotropic drug (ATC codes: N05A-C, N06A) at a participating primary care centre. All patients and BHC appointments were identified through their medical records. Exclusion criteria included patients younger than 18 years or older than 24 years to enable a focus on the critical young adults group.

### Outcomes

Access to mental health services was measured as utilization (outcomes 1 and 2) and waiting times (outcomes 3 and 4) as follows:

1. Number of unique individual patients: total number of unique individuals with in-person or remote appointments with a BHC at a participating primary care centre.
2. Number of appointments by all patients: total number of in-person and remote appointments with a BHC at a participating primary care centre.
3. Average waiting time for all appointments: average waiting time in days for all appointments (both first appointments and further appointments) with a BHC at a participating primary care centre.
4. Average waiting time for first appointment: average waiting time in days from the patient’s initial contact with a participating primary care centre to the patient’s first appointment with a BHC at that centre.

Together, these four measures constitute the study’s operational definition of the PCBH model’s impact on access to mental health services.

All appointments with BHCs at the participating centres were included, encompassing both in-person and remote appointments. In-person appointments were categorized into three types: (1) individual patient appointments with BHCs; (2) group session appointments involving multiple patients’ appointments with BHCs; and (3) primary care team appointments, i.e. patients’ appointments with a team of primary care providers. Remote appointments covered both regular phone calls and digital appointments such as video calls and internet-based cognitive behavioural therapy via secure digital tools at Region Östergötland’s digtal platform.

### Data analysis

Statistical analyses were conducted using Stata v. 17.0 (StataCorp LLC, College Station, TX, USA), with the significance level set at *p* < 0.05. Data from electronic medical records were collected and directly transferred from the computerized system of Region Östergötland into Stata software. The waiting time for the first appointment was calculated from the initial contact with the PCC by an individual meeting the inclusion criteria to their first in-person appointment with a physician or BHC. The overall waiting time for appointments was calculated as the mean waiting time for all in-person visits during each period. During data processing, the first visit each year for all included individuals was considered a new visit, and all subsequent visits that year were relabeled as follow-up visits. This was justified due to clinical experience where the clinical average episode of care is < 1 year.

Comparisons between different time periods regarding waiting times (mean waiting times and waiting times for the first appointment) were tested using one-way ANOVA, with multiple comparisons adjusted using the Sidak correction. Outliers, defined as waiting times exceeding 100 days, were excluded from the analyses to reduce skewness. Waiting times below 0 days were interpreted as coding errors and also excluded from the analyses. Differences between in the number of appointments with the BHC between time periods were analysed using a z test (two-sample test of proportions), with adjustments for multiple comparisons made using Bonferroni correction. Initially, all appointment types were analysed together, followed by sub-analyses for on-site and remote appointments separately. All centres were analyzed together, i.e. not divided into two clusters.

## Results

Utilization data, including the number of unique patients and the number of appointments, are summarized in Table 3, and the average waiting times, covering both all appointments and first-time appointments, are presented in Table 4.

**Table 3:** Utilization: number of individual patients and BHC appointments at the participating primary care centres

Appointments and patients	Pre-facilitation (year 1)	Facilitation (year 3)		Post-facilitation (year 4)		
	Number	Number	*p* value compared with pre-facilitation	Number	*p* value compared with facilitation	*p* value compared with pre-facilitation
**Number of unique individual patients**	67	123	< 0.001	84	< 0.001	0.20
**Number of appointments**	1135	1318	< 0.001	1279	0.88	0.006
**In-person appointments**						
Total	796	785	0.78	828	0.28	0.43
- Individual patient appointments	782	773	> 0.99	826	0.19	0.27
- Group session appointments	6	9	> 0.99	2	0.10	0.16
- Primary care team appointments	8	3	> 0.99	0	0.08	0.005
**Remote appointments**						
Total	339	533	< 0.001	451	0.01	0.014
- Phone appointments	10	126	< 0.001	117	0.56	< 0.001
- Digital appointments	329	407	0.004	334	0.007	0.005

Patients were aged 18–24 years.

**Table 4:** Average waiting times for in-person appointments with a BHC by patients aged 18–24 years

Appointments	Waiting time (days), mean (SD) (*n*)					
	Pre-facilitation (year 1)	Facilitation (year 3)	*p* value compared with pre-facilitation	Post-facilitation (year 4)	*p* value compared with facilitation	*p* value compared with pre-facilitation
Appointments						
**All appointments**	14.1 (13.9) (*n* = 1135)	11.8 (11.7) (*n* = 1318)	**< 0.001**	13.4 (13.0) (*n* = 1279)	0.39	0.007
All individual in-person appointments	15.3 (13.2) (*n* = 782)	13.0 (10.5) (*n* = 773)	**< 0.001**	13.8 (11.6) (*n* = 826)	0.46	**< 0.001**
All individual remote appointments	11.4 (15.1) (*n* = 353)	10.0 (12.9) (*n* = 545)	0.45	12.5 (15.3) (*n* = 453)	**0.022**	0.62
**First appointments**						
All first appointments	8.8 (10.4) (*n* = 67)	6.8 (6.6) (*n* = 123)	0.24	6.8 (6.0) (*n* = 84)	> 0.99	0.28
All in-person first appointments	8.8 (10.4) (*n* = 66)	7.1 (6.4) (*n* = 114)	**< 0.001**	7.1 (6.1) (*n* = 77)	> 0.99	**< 0.001**

BHC, behavioral health consultant; SD, standard deviation. Significance results are in bold type.

### Number of unique individual patients

The number of unique individual patients aged 18–24 years who had a BHC appointment, both in-person and remote, increased from 67 during the pre-facilitation to 123 during the facilitation phase, i.e. an 84% increase from the pre-facilitation baseline (*p* < 0.001). However, in the year after facilitation (the post-facilitation phase), the number of individual patients decreased to 84 (*p* < 0.001). This reflects a 25% increase from pre-facilitation, a change that was not statistically significant (*p* = 0.20). The decline in the number of patients from the facilitation phase to the post-facilitation phase was significant (*p* < 0.001).

### Number of appointments by all patients

The number of appointments with a BHC for individuals aged 18–24 years, encompassing both in-person and remote appointments, increased from 1135 at the pre-facilitation baseline to 1318 during the facilitation phase, a 16% increase from baseline (*p* < 0.001). In the post-facilitation phase, the number of appointments decreased slightly to 1279, which was a 13% increase from the pre-facilitation baseline (*p* = 0.006). There was no significant difference in the proportions between the facilitation and post-facilitation phases, indicating that appointment numbers remained stable during and after facilitation (*p* = 0.88).

Sub-analyses including all the in-person appointments revealed no significant differences in the number of appointments at the participating primary care centres between pre-facilitation and the facilitation phase (*p* > 0.99). Similarly, there was no significant difference between the pre-facilitation baseline and the post-facilitation phase (*p* = 0.54).

In the sub-analysis of all remote appointments, significant differences were observed in the number of appointments at participating primary care centres between the pre-facilitation baseline (*n* = 339) and the facilitation phase (*n* = 533; *p* < 0.001). Similarly, a significant difference was found between the pre-facilitation baseline and the post-facilitation phase (*n* = 451; *p* = 0.01). In addition, there was a significant difference between the facilitation and post-facilitation phases (*p* = 0.014).

### Average waiting time for all appointments

The average waiting time for all appointments with a BHC for those aged 18–24 years decreased during the facilitation and post-facilitation phases compared with the pre-facilitation baseline. Specifically, the waiting time reduced from 14.1 days at pre-facilitation to 11.8 days during the facilitation phase (*p* < 0.001 compared with the pre-facilitation baseline) and then increased slightly to 13.4 days in the post-facilitation phase (*p* = 0.39 compared with the facilitation phase). The decrease in waiting time from the pre-facilitation baseline to the post-facilitation phase was significant (*p* = 0.007).

Similar trends were observed in sub-analyses focused solely on individual in-person appointments. The average waiting time decreased from 15.3 days at the pre-facilitation baseline to 13.0 days during the facilitation phase (*p* < 0.001 compared with the pre-facilitation baseline) and then increased slightly to 13.8 days in the post-facilitation phase (*p* = 0.46 compared with the facilitation phase). The decrease in waiting time from the pre-facilitation baseline to the post-facilitation phase was significant (*p* < 0.001).

Sub-analysis of remote appointments indicated that the average waiting time decreased non-significantly from 11.4 days at the pre-facilitation baseline to 10.0 days during the facilitation phase (*p* = 0.45 compared with the pre-facilitation baseline). In the post-facilitation phase, the average waiting time increased to 12.5 days, a significant increase compared with the facilitation phase. There was no significant difference between the pre- and post-facilitation phases (*p* = 0.62).

### Average waiting time for the first appointment

When focusing on waiting times for the first appointment with a BHC, a non-significant reduction in waiting times was found during the facilitation and post-facilitation phases compared with the pre-facilitation baseline. Specifically, the waiting time for the first appointment decreased from 8.8 days at the pre-facilitation baseline to 6.8 days both during facilitation (*p* = 0.24) and after it (*p* = 0.28).

Similar results were observed when analysing in-person first appointments only. The average waiting time declined from 8.8 days at the pre-facilitation baseline to 7.1 days both during and post-facilitation (*p* < 0.001 for both comparisons compared with the pre-facilitation baseline).

Regarding remote appointments, only one individual had a first remote appointment with a BHC in the pre-facilitation phase, and seven individuals had a first remote appointment in the facilitation and post-facilitation phases; hence, the small sample size was deemed insufficient to conduct meaningful statistical analyses.

## Discussion

This study evaluated the impact of integrating the PCBH service delivery model into Swedish primary care mental health services provided by BHCs to young adults aged 18–24 years with regard to number of patients and appointments as well as waiting times for these BHC appointments. The PCBH model reorganizes primary care by embedding BHCs into teams, optimizing resources while improving access, efficiency, and mental health care quality. The integration of the model into routine operations was facilitated over one year. We found that there was an increase in the number of individual patients utilizing mental health care services, as well as an increase in the total number of appointments with the BHCs. In addition, waiting times for in-person appointments with a BHC were reduced, benefiting both the average waiting times and waiting times for first appointments. These findings are in line with the aim of the PCBH model to provide accessible mental health services to a larger section of the population. However, it is important to note that the lack of a comparison group precludes definitive conclusions regarding the effects of the model, as alternative explanations for the observed changes cannot be ruled out.

The encouraging results regarding both the utilization of mental health services and waiting times align with previous research on the PCBH model. An emerging evidence base supports the effectiveness of PCBH and elucidates its mechanisms of change [25]. A review of 35 studies found that PCBH was associated with shorter waiting times for treatment, a higher likelihood of patient engagement in behavioural health services, and an increased number of appointments attended [26]. However, this review focused primarily on adult and elderly populations, as well as specific groups such as war veterans. Our findings contribute to this body of literature by specifically examining young adults’ access to and the impact of PCBH within a community population.

Most parameters revealed a decline in impact in the year after the integration of the PCBH model into routine operations, suggesting that the most substantial effects were achieved during the year when the integration of the model was facilitated by an implementation team and regional facilitators, with a gradual decrease in the subsequent year. This trend suggests that sustained implementation support may be important for long-term impact. It also underscores the necessity for further research into the long-term application of PCBH. Once the novelty diminishes, it becomes crucial to examine how well the PCBH components are being integrated into routine primary care operations.

Identifying the optimal timing for measuring service outcomes presents significant challenges [27]. On the one hand, research indicates that implementation processes require time, suggesting that longitudinal study designs are necessary to capture a more accurate picture of outcomes. Conversely, other studies reveal that substantial effects are often observed in the short term, particularly when new practices are first introduced, accompanied by robust support for integrating these practices into routine operations [28]. This novelty effect may arise from the increased enthusiasm associated with adopting new approaches, which can drive efforts that may not be sustainable in the long term [29, 30]. Whether this applies to the PCBH model remains unknown, as no long-term studies have yet been conducted.

The way in which the PCBH model is integrated into routine operations likely has an impact on the accessibility to mental health services offered by BHCs. In our study, the model was introduced with the assistance of local multi-professional implementation teams at each primary care centre, alongside external regional facilitators who supported all participating centres throughout the facilitation phase. These teams primarily acted as intermediaries between staff members and regional facilitators. In this capacity, they may have functioned as informal change agents, as defined in Rogers’ Diffusion of Innovations theory, whereby such individuals promote and facilitate the adoption of new ideas or practices within a social system [31]. Change agents typically play a crucial role in influencing the attitudes and behaviours of others, often leveraging their credibility and knowledge to encourage the adoption of new practices [31]. The importance of this type of intermediary is emphasized in many of the theories, models and frameworks used in implementation science [23]. The on-site implementation teams were likely important for building trust within the primary care centres, helping to alleviate any scepticism and resistance that might have existed.

Methodological considerations must be accounted for when interpreting the findings. The study was conducted in nine primary care centres in a single region of Sweden, which may limit the generalizability of the results to other health care contexts and systems. However, the size of the primary care centres varied considerably, and the centres were located in a mix of urban and rural areas, as well as low and high socio-economic areas. These variations are important for generalizability. Larger centres typically have more resources, including staff and facilities, which can have an impact on the range of services offered and their ability to meet patients’ mental health needs. In contrast, smaller centres may face challenges in providing the same level of care or access to mental health services. In addition, the patient populations served by larger centres often differ markedly from those in smaller centres, influencing the types of mental health issues encountered and the approaches to addressing them. These differences enhance the applicability of findings within Swedishprimary care settings.

Another methodological issue of concern is the potential influence of the COVID-19 pandemic on the results. The pre-facilitation phase (May 2017 to November 2018) occurred before the pandemic, whereas the facilitation phase (May 2019 to November 2020) partially overlapped with it. The post-facilitation phase (May 2020 to November 2021) took place entirely during the pandemic. Sweden experienced a significant decline in the total number of primary care consultations during the pandemic, estimated to be around 12% lower in 2020 compared with 2019 [32]. Concurrently, remote consultations, encompassing communication via digital tools, telephone interactions and written communications, increased from approximately 12% of total consultations before the pandemic to about 17% during this period [32].

In a broader context, Sweden’s experience mirrored trends observed in other countries. The INTRePID study, which surveyed nine countries including Sweden, found that although many countries saw a substantial decline in primary care visits during the pandemic, Sweden’s overall volume of visits remained relatively stable [33]. This apparent discrepancy may be explained by the increase in remote consultations, which likely compensated for some of the decline in face-to-face visits. However, mental health issues appeared to escalate during the pandemic years. Before the pandemic, the prevalence of current depressive disorder in Sweden was estimated at 8.75% [34]. During the pandemic, 15.6% of Swedes reported experiencing significant symptoms of depression, and 9.5% reported symptoms of anxiety [35]. These findings suggest that while overall primary care consultations decreased, the burden of mental health concerns increased during this time.

A key methodological limitation of the study is that it did not analyse the specific PCBH services delivered, such as brief interventions or other evidence-based treatments for particular diagnoses, nor did it evaluate the effects of these appointments on patient outcomes. Consequently, the study cannot conclude improvements in patient functioning or symptom remission resulting from the integration of the PCBH model into routine care. A recent study has shown promising results on the effectiveness of brief interventions, typically provided within PCBH, among several clinical populations [36], and future research should address these gaps for the young adult population to better understand the potential benefits of the model for patients with mental health concerns. In addition, examining implementation outcomes such as fidelity with core components and sustainability, as well as the model’s cost-effectiveness, is essential. The study did not examine potential ripple effects, i.e. unintended or indirect consequences extending beyond the intended impact of the PCBH model on patients presenting to BHCs, nor did it assess effects on other patient groups within primary care. Finally, the study was not pre-registered as a clinical trial.

Next, several strengths can be noted about this study. It utilizes robust data from medical records with a pragmatic stepped-wedge cluster trial design. By examining various primary care centres, the study provided a comprehensive understanding of the impact of the PCBH model on young adults’ access to mental health care. Including multiple cases enhances the generalizability of the findings across different contexts. Data were collected at multiple time points, both before and after the integration of the model into routine operations. This approach allowed for a thorough assessment of trends over time and identification of any changes associated with the model. Although some of the changes are modest and the clinical relevance for overall appointment waiting times remains uncertain, the data consistently suggest that more patients are receiving care in a timelier manner.

Overall, this study addresses an important research gap by evaluating the integration of the PCBH model into routine primary care operations. In addition, its focus on a young adult population is crucial because much of the existing PCBH research has primarily targeted specific health conditions or narrowly defined groups. This study tackles the challenges faced by primary care in providing timely and effective mental health care to young individuals.

In conclusion, integrating the PCBH model into routine Swedish primary care significantly improved access to mental health services for young adults, as shown by increased utilization and reduced waiting times. The findings highlight the model’s potential for scalable, team-based mental health delivery in primary care. However, declines post-facilitation suggest that sustained implementation support may be important for long-term impact. It should be noted that the lack of a comparison group precludes definitive conclusions regarding the effects of the model, as alternative explanations for the observed changes cannot be ruled out.

## Data Availability

The authors confirm that all data supporting the findings of this study are available within the paper.

## Abbreviations

ATC: Anatomical Therapeutic Chemical
BHC: Behavioural Health Consultant
EMR: Electronic Medical Records
ICD-10: International Statistical Classification of Diseases and Related Health Problems 10th Revision
OECD: The Organization for Economic Co-operation and Development
PCBH: Primary Care Behavioral Health
PCC: primary care centers
